# A Competency-based Tool for Resident Evaluation of Pediatric Emergency Department Faculty

**DOI:** 10.5811/westjem.2022.11.57686

**Published:** 2022-12-21

**Authors:** Ethan Sandler, Michael Lee, Rebecca Carter, Natalie L. Davis, Sarah B. Dubbs, Karen Webster, Katelyn E. Donohue

**Affiliations:** * University of Maryland School of Medicine, Department of Pediatrics, Baltimore, Maryland; † University of Maryland School of Medicine, Baltimore, Maryland; ‡ University of Maryland School of Medicine, Division of General Pediatrics, Department of Pediatrics, Baltimore, Maryland; § University of Maryland School of Medicine, Division of Neonatology, Department of Pediatrics, Baltimore, Maryland; || University of Maryland School of Medicine, Department of Emergency Medicine, Baltimore, Maryland; # University of Maryland School of Medicine, Divisions of Pediatric Emergency Medicine and General Internal Medicine, Departments of Pediatrics and Internal Medicine, Baltimore, Maryland

## BACKGROUND

The quality of teaching skills among faculty is critical to allowing trainees to gain competence for independent practice, and while considerable progress has been made on trainee assessment, faculty evaluation tools have lagged behind.^1^–^3^ The Accreditation Council for Graduate Medical Education (ACGME) Common Program Requirements dictate that residents must evaluate faculty, underscoring the need for quality evaluation tools.^4^,^5^ A competency-based evaluation (CBE) emphasizes behaviorally focused skills and developmental outcomes, and has long been used to assess trainees; however, there are no published CBEs for faculty in pediatric emergency medicine (PEM), and limited tools in pediatrics and EM.^6^–^9^

The ACGME has established six core physician competencies that are the gold standard in graduate medical education and continue to be developed into specialty-specific CBEs with “Pediatric Milestones 2.0” and “Emergency Medicine Milestones 2.0.”^4^,^5^,^10^–^12^ To address the lack of faculty assessments in PEM, we aimed to develop a specific CBE using the conceptual framework of the ACGME milestones, with behavioral anchors incorporating previously published tools, such as the Stanford Faculty Development Program.^13^–^16^

## OBJECTIVES

Our objectives were to develop a CBE tool 1) for formative assessment on pediatric emergency department (PED)-specific teaching skills, including procedural instruction, 2) that trainees perceive as efficient and effective, and 3) that faculty find useful for their development as educators.

## CURRICULAR DESIGN

The CBE was designed using iterative review by a cohort of six clinician-educators from the departments of pediatrics and emergency medicine, including graduates of the Harvard Macy Institute, American College of Emergency Physicians teaching fellowship, and the Johns Hopkins University Master of Education in the Health Professions. The cohort members all have experience on clinical competency committees, and in faculty and program evaluation. For content validity, we conducted a literature review of existing tools, and through consensus methodology we identified skills critical to a PED attending and developed sub-competencies from the ACGME core competencies. Behavioral anchors were adapted from a CBE used for general surgery faculty, the Pediatric and Emergency Medicine Milestones, and the Stanford Faculty Development tool.^2^,^4^,^5^,^14^ We used a milestone scale, with half-steps to indicate that the lower milestone has been demonstrated, as well as some skills of the higher milestone. The tool was then evaluated by additional educational reviewers beyond the initial cohort to ensure efficiency of use and readability, and to consider whether critical items had not yet been included. The review process resulted in 11 sub-competencies distributed over the ACGME competencies (Table).

Trainees completed both an existing Likert assessment without behavioral anchors and the CBEs over a six-month period. Trainees and faculty were surveyed on use of the CBE tool for efficacy, efficiency, and satisfaction to determine Kirkpatrick Level 1 (reactions) outcome attainment at the conclusion of the pilot period.^17^ No faculty or residents who completed the surveys were involved in the development of the tool. This initiative was deemed exempt by the University of Maryland Institutional Review Board.

## IMPACT/EFFECTIVENESS

A total of 143 CBEs of seven PED faculty were assigned, and trainees completed all assigned evaluations. All faculty (7), and 45% of residents (17) responded to the survey; survey items were rated on a five-point scale. Primarily pediatrics trainees completed the survey (10) and included both intern (10) and senior (7) trainees. The CBE tool was rated by 71% of residents as easy to understand (mean 3.6, SD 0.6), and 76% agreed or strongly agreed the CBE allowed them to effectively evaluate faculty (mean 3.9, SD 0.6). Most residents agreed or strongly agreed they are satisfied with the CBE (mean 3.8, SD 0.6), with no residents disagreeing. After reviewing six months of their CBEs, 71% of faculty reported the tool was formative (mean 4.3, SD 1), and 86% felt it was easy to understand (mean 4.4, SD 0.8). Importantly, 86% of faculty agreed with the areas for improvement identified (mean 4.4, SD 0.8).

The CBE was longer than the previous Likert evaluation; however, trainees felt the CBE remained efficient. The milestone scales with behavioral anchors and half-steps were intended to indicate when faculty were between milestones. While this mirrors the design of the ACGME milestones, it may have created the opportunity for personal bias to affect the CBE.^4^,^5^ Additional limitations include our use of a small sample size of faculty, trainees from multiple specialties with possible different expectations of faculty, and a lower response rate on trainee surveys.

Importantly, our CBE tool introduced two sub-competency items addressing technical skills and procedural autonomy. The use of procedural-focused faculty competencies is unique, with only two previously published items, one within general surgery, and one within an EM shift-based feedback tool.^2^,^6^ Procedures are a significant component of PEM and a critical area of assessment for faculty. Procedural autonomy is of critical importance to trainees’ development; however, we acknowledge that a trainee’s opinion of how much autonomy they should be granted is biased and makes interpretation of this competency more challenging.^18^–^20^

This PED CBE could be adapted to other clinical teaching experiences, with the caveat that there is likely variation between different specialties regarding teaching expectations. Our future goals are to assess reliability after a full year of implementation and investigate the role of CBEs in departmental educational offerings for faculty. The ultimate goal is improvement in faculty teaching behaviors, progressing to Kirkpatrick Level 3 (behavior) outcomes.^17^

There are important considerations for other programs hoping to implement faculty CBEs. As the goal of the CBE is for individual skill development, faculty buy-in is critical to successful implementation. Faculty were briefed on the change prior to implementation and were educated on the role of CBEs in professional development. Notably, this assessment is from the perspective of trainees and must be paired with direct observation, and peer and supervisor evaluations to create a complete assessment of a teaching competency. Additionally, it is important to consider the limitations of the evaluation management system when developing a CBE, as the system needs to support behavioral anchor descriptions.

In summary, this was an impactful and feasible intervention of a faculty competency-based evaluation in our pediatric emergency department, including two new procedural sub-competencies, that was well received by trainees and faculty members.

## Supplementary Information





## Figures and Tables

**Table:** Competency-based evaluation of pediatric emergency department.

Critical deficiency – Level 1	Variable skills – Level 2	Effective skills – Level 3	Exemplary skills – Level 4
Procedural Autonomy: Balances supervision and autonomy			
Permits no or very limited procedural autonomy for the level of training. Residents are rarely allowed to attempt common pediatric emergency procedures.	Allows for simple involvement with procedures, attempts to allow participation based on skill level.	Provides residents with appropriate procedural supervision commensurate with their level of training and promotes progressive procedural autonomy.	Encourages proficient residents to teach procedural skills to others, works with residents to develop procedural independence, encourages residents to take on challenging procedures.

Patient Care Autonomy: Balances supervision and autonomy			

Permits no or very limited autonomy. Residents are rarely allowed to practice independent decision making appropriate for their level of training.	Allows residents to see patient and make care decisions appropriate for level of training but always reviews prior to implementation. Limits independent decision making and senior supervision of interns.	Provides resident with appropriate supervision commensurate with their level of training, endorses progressive autonomy, and allows senior residents to guide interns.	Expects senior residents to manage complex patients independently with immediate availability and encourages interns to take on increasingly complex patients. All residents are encouraged to develop and implement care decisions.

Knowledge Base: Promotes understanding of knowledge and use of clinical reasoning			

Rigid or outdated approach to clinical scenarios.	Demonstrates knowledge of several approaches used in the field. Able to describe the benefits of their own approach.	Up to date on emerging research in their field. Aware of the utility of novel approaches. Discusses patient-specific data in the context of clinical decision making.	Encourages and assists residents to become up to date on relevant literature. Dedicates time to synthesize emerging data and discuss how to apply it to patient care. Coaches residents to use relevant literature in their clinical decision making.

Technical Skills: Demonstrates technical skill with procedures.			

Does not perform rare or time-sensitive procedures with consistent success. Does not coach residents to perform procedures or does not provide sufficient procedural supervision.	Performs rare or time- sensitive procedures with accuracy. Limited coaching for residents to perform procedures, minimal educating on procedural complications.	Able to perform rare or time- sensitive procedures with accuracy. Coaches residents to perform simple procedures. Educates on common procedural complications.	Able to perform rare or time- sensitive advanced procedures with accuracy and efficiency. Coaches residents to perform both simple and advanced procedures with good technique and educates on common and rare complications.

Evidence-based Medicine: Promotes the use of EBM in clinical practice			

Does not use evidence-based medicine.	Conceptually supports using evidence-based medicine but is limited in implementing new approaches into clinical practice.	Readily identifies studies that support their approach to patient care. Discusses new evidence and its impact on their current practice.	Discusses up-to-date studies that impact patient care with residents. Seamlessly adapts clinical practice to incorporate new approaches when appropriate. Teaches residents how to use EBM themselves.

Feedback: Provides formative feedback			

Provides little or no feedback of any type.	Offers generalized feedback consisting mostly of positive reinforcement. Provides little to no corrective or constructive feedback.	Provides timely corrective and constructive feedback and positive reinforcement. Corrective feedback accompanied by practical suggestions for improvement.	Frequent corrective and constructive feedback with explanations. Able to adjust feedback based on resident needs to foster self-motivated learning and implementation of suggestions for improvement.

Team Dynamics: Works in interprofessional teams to enhance patient safety and improve patient care quality			

Develops care plans independently of the rest of the team. Limited involvement of the patient and family in shared decision making. Rarely uses consultants or provides a minimal level of communication with consultants.	Uses consultants and support services in developing care plans. Involves patients, families, and residents in the plan of care with some opportunities for shared decision making.	Uses consultants and support services in developing care plans and encourages residents to do the same. Involves residents, patients, and families in shared decision making and solicits feedback from families.	Works with consultants, support services, residents, and families effectively to use shared decision making. Coaches residents to communicate effectively with consultants and support services to improve patient care. Coaches residents on how to develop shared decision making with patients and families.

Leadership: Demonstrates leadership skills and encourages residents to take on leadership roles in PED			

Does not demonstrate effective leadership in most situations. Does not teach residents about effective leadership skills.	Demonstrates leadership in most situations but at times may be noticeably uncomfortable. Does not discuss the importance of leadership or how to effectively lead a team with residents.	Demonstrates exemplary leadership in their area of expertise but can satisfactorily lead team in all situations. Teaches residents effective leadership skills but does not always encourage them to assume leadership roles themselves.	Demonstrates exemplary leadership skills in both emergent and non-emergent situations. Encourages residents to assume leaderships roles in the ED that are appropriate to their level of training.

Cultural Sensitivity: Demonstrates and promotes cultural sensitivity			

Frequently lacks cultural sensitivity or responds uniformly to patients regardless of diverse backgrounds. Does not coach or educate residents to demonstrate cultural sensitivity.	Demonstrates sensitivity and responsiveness to diverse populations in most situations. Does not coach or educate residents to demonstrate cultural sensitivity.	Demonstrates sensitivity and responsiveness to diverse populations in all settings, including but not limited to diversity in gender, age, culture, race, religion, disabilities, and sexual orientation.	Demonstrates sensitivity and responsiveness to patients in all situations, including but not limited to diversity in gender, age, culture, race, religion, disabilities, and sexual orientation. Coaches residents to demonstrate the same level of cultural sensitivity.

Communication: Promotes effective communication with patients, families, and other health professionals			

Uses standard medical interview template to engage all patients regardless of unique socioeconomic, cultural, and physical needs. Does not effectively engage other health professionals.	Attempts to identify unique aspects of each patient and use them to establish an effective physician-patient alliance. Approaches all healthcare professionals in the same way, regardless of their role in patient care.	Systematically identifies the unique needs of each patient and uses them to build a strong physician-patient relationship. Effectively communicates with other healthcare professionals with an understanding of their role in patient care.	Effortlessly identifies the unique needs of each patient and builds an authentic relationship with them and their support system. Seamlessly broaches sensitive topics in a way that puts patients at ease. Approaches other healthcare professionals as individuals to build a working relationship that provides the best outcomes for the patient.

Teaching Style: Establishes positive learning climate			

Performs little education, does not encourage resident participation in academic discussions.	Performs didactic teaching but teaching sessions are not tailored to resident’s level of training. May ask for resident opinions with limited discussion.	Solicits resident opinions and discusses their merits on a basic level. Willing to teach complex topics. Tailors teaching to resident’s level of training. Provides guidance of future topics to study.	Encourages residents to share opinions and provide individualized teaching based on resident competency level. Provides the tools and motivation necessary for residents to formulate essential questions and to self-teach complex topics.

*PED*, pediatric emergency department; *EBM*, evidence-based medicine
