# Supplementary material for: A Competency-based Tool for Resident Evaluation of Pediatric Emergency Department Faculty
Source: West J Emerg Med. 2022 Dec 21;24(1):59–63. doi: 10.5811/westjem.2022.11.57686 (PMC9897249; doi:10.5811/westjem.2022.11.57686)
Supplement: Supplementary file 1 [file wjem-24-59-s001.docx]

**Appendix 1.** Likert Scale Faculty Evaluation Tool

| Evaluation Item | Does not meet my expectations | Meets my expectations | | Exceeds my expectations | | One of the best role models for this competency |  |
| --- | --- | --- | --- | --- | --- | --- | --- |
| 1. Models patient-centered care by considering patient preferences and shared decision making with the patient/family as appropriate |  |  |  | |  | | |
| 1. Provides appropriate guidance and supervision to the team in the care of patients based on extensive background knowledge |  |  |  | |  | | |
| 1. Models the importance of life-long learning by participating with the team in accessing up to date scientific evidence that is needed to care for patients. |  |  |  | |  | | |
| 1. Emphasizes the importance of ongoing resident education by encouraging resident conference attendance. |  |  |  | |  | | |
| 1. Creates an environment that is conducive to learning |  |  |  | |  | | |
| 1. Demonstrates the ability to communicate with patients and families in a way that puts them at ease and encourages them to be open about their questions and concerns. |  |  |  | |  | | |
| 1. Actively engages in a leadership role that facilitates team communication and team function |  |  |  | |  | | |
| 1. Models professionalism by demonstrating respect, altruism, integrity, and responsibility in interactions with patients/families, staff, colleagues and learners |  |  |  | |  | | |
| 1. Easily accessible and welcomes the opportunity to answer questions, teach, and provide guidance in patient care |  |  |  | |  | | |
| 1. Models ethical behavior and sensitivity to cultural diversity when working with the health care team to provide care to patients. |  |  |  | |  | | |
| 1. Discusses resources, costs, and impact of diagnostic tests/therapeutic modalities in advocating for the care of patients. |  |  |  | |  | | |
| 1. Establishes rapport with the health care team by acknowledging the importance of each member's contributions to the care of the patient and providing appropriate guidance balanced with autonomy |  |  |  | |  | | |
