# Supplementary material for: A Competency-based Tool for Resident Evaluation of Pediatric Emergency Department Faculty
Source: West J Emerg Med. 2022 Dec 21;24(1):59–63. doi: 10.5811/westjem.2022.11.57686 (PMC9897249; doi:10.5811/westjem.2022.11.57686)
Supplement: Supplementary file 2 [file wjem-24-59-s002.docx]

**Appendix 2.** Competency Based Faculty Evaluation Tool

Procedural Autonomy: Balances supervision and autonomy

| Critical Deficiency – Level 1 | Variable Skills –  Level 2 | Effective Skills – Level 3 | Exemplary Skills – Level 4 |
| --- | --- | --- | --- |
| Permits no or very  limited procedural autonomy for the level of training. Residents are rarely allowed to attempt common pediatric emergency procedures. | Allows for simple involvement with procedures, attempts to allow participation based on skill level. | Provides residents with appropriate procedural supervision commensurate with their level of training, and promotes progressive procedural autonomy. | Encourages proficient residents to teach procedural skills to others, works with residents to develop procedural independence, encourages residents to take on challenging procedures. |

Patient Care Autonomy: Balances supervision and autonomy

| Critical Deficiency – Level 1 | Variable Skills –  Level 2 | Effective Skills – Level 3 | Exemplary Skills – Level 4 |
| --- | --- | --- | --- |
| Permits no or very limited autonomy. Residents are rarely allowed to practice independent decision making appropriate for their level of training. | Allows residents to see patient and make care decisions appropriate for level of training but always reviews prior to implementation. Limits independent decision making and senior supervision of interns. | Provides resident with appropriate supervision commensurate with their level of training, endorses progressive autonomy, and allows senior residents to guide interns. | Expects senior residents to manage complex patients independently with immediate availability and encourages interns to take on increasingly complex patients. All residents are encouraged to develop and implement care decisions. |

Knowledge Base: Promotes understanding of knowledge and use of clinical reasoning

| Critical Deficiency – Level 1 | Variable Skills –  Level 2 | Effective Skills – Level 3 | Exemplary Skills – Level 4 |
| --- | --- | --- | --- |
| Rigid or outdated approach to clinical scenarios. | Demonstrates knowledge of several approaches utilized in the field. Able to describe the benefits of their own approach. | Up to date on emerging research in their field. Aware of the utility of novel approaches. Discusses patient specific data in the context of clinical decision making. | Encourages and assists residents to become up to date on relevant literature. Dedicates time to synthesize emerging data and discuss how to apply it to patient care. Coaches residents to use relevant literature in their clinical decision making. |

Technical Skills: Demonstrates technical skill with procedures.

| Critical Deficiency – Level 1 | Variable Skills –  Level 2 | Effective Skills –  Level 3 | Exemplary Skills – Level 4 |
| --- | --- | --- | --- |
| Does not perform rare or time sensitive procedures with consistent success. Does not coach residents to perform procedures or does not provide sufficient procedural supervision. | Performs rare or time sensitive procedures with accuracy. Limited coaching for residents to perform procedures, minimal educating on procedural complications. | Able to perform rare or time sensitive procedures with accuracy. Coaches residents to perform simple procedures. Educates on common procedural complications. | Able to perform rare or time sensitive advanced procedures with accuracy and efficiency. Coaches residents to perform both simple and advanced procedures with good technique and educates on common and rare complications. |

Evidence Based Medicine: Promotes the use of EBM in clinical practice

| Critical Deficiency – Level 1 | Variable Skills –  Level 2 | Effective Skills –  Level 3 | Exemplary Skills – Level 4 |
| --- | --- | --- | --- |
| Does not utilize evidence-based medicine. | Conceptually supports utilizing evidence-based medicine but is limited in implementing new approaches into clinical practice. | Readily identifies studies that support their approach to patient care. Discusses new evidence and its impact on their current practice. | Discusses up to date studies that impact patient care with residents. Seamlessly adapts clinical practice to incorporate new approaches when appropriate. Teaches residents how to utilize EBM themselves. |

Feedback: Provides formative feedback

| Critical Deficiency – Level 1 | Variable Skills –  Level 2 | Effective Skills –  Level 3 | Exemplary Skills – Level 4 |
| --- | --- | --- | --- |
| Provides little or no feedback of any type. | Offers generalized feedback consisting mostly of positive reinforcement. Provides little to no corrective or constructive feedback. | Provides timely corrective and constructive feedback and positive reinforcement. Corrective feedback accompanied by practical suggestions for improvement. | Frequent corrective and constructive feedback with explanations. Able to adjust feedback based on resident needs to foster self-motivated learning and implementation of suggestions for improvement. |

Team Dynamics: work in interprofessional teams to enhance patient safety and improve patient care quality

| Critical Deficiency – Level 1 | Variable Skills –  Level 2 | Effective Skills –  Level 3 | Exemplary Skills – Level 4 |
| --- | --- | --- | --- |
| Develops care plans independently of the rest of the team. Limited involvement of the patient and family in shared decision making. Rarely utilizes consultants or provides a minimal level of communication with consultants. | Utilizes consultants and support services in developing care plans. Involves patients, families residents in the plan of care with some opportunities for shared decision making. | Utilizes consultants and support services in developing care plans and encourages residents to do the same. Involves residents, patients and families in shared decision making and solicits feedback from families. | Works with consultants, support services, residents and families effectively to utilize shared decision making. Coaches residents to communicate effectively with consultants and support services to improve patient care. Coaches residents on how to develop shared decision making with patients and families. |

Leadership: Demonstrates leadership skills and encourages residents to take on leadership roles in PED

| Critical Deficiency – Level 1 | Variable Skills –  Level 2 | Effective Skills –  Level 3 | Exemplary Skills – Level 4 |
| --- | --- | --- | --- |
| Does not demonstrate effective leadership in most situations. Does not teach residents about effective leadership skills. | Demonstrates leadership in most situations but at times may be noticeably uncomfortable. Does not discuss the importance of leadership or how to effectively lead a team with residents. | Demonstrates exemplary leadership in their area of expertise but can satisfactorily lead team in all situations. Teaches residents effective leadership skills but does not always encourage them to assume leadership roles themselves. | Demonstrates exemplary leadership skills in both emergent and non-emergent situations. Encourages residents to assume leaderships roles in the Emergency room that are appropriate to their level of training. |

Cultural Sensitivity: Demonstrates and promotes cultural sensitivity

| Critical Deficiency – Level 1 | Variable Skills –  Level 2 | Effective Skills –  Level 3 | Exemplary Skills – Level 4 |
| --- | --- | --- | --- |
| Frequently lacks cultural sensitivity, or responds uniformly to patients regardless of diverse backgrounds. Does not coach or educate residents to demonstrate cultural sensitivity. | Demonstrates sensitivity and responsiveness to diverse populations in most situations. Does not coach or educate residents to demonstrate cultural sensitivity. | Demonstrates sensitivity and responsiveness to diverse populations in all settings, including but not limited to diversity in gender, age, culture, race, religion, disabilities, and sexual orientation. | Demonstrates sensitivity and responsiveness to patients in all situations, including but not limited to diversity in gender, age, culture, race, religion, disabilities, and sexual orientation. Coaches residents to demonstrate the same level of cultural sensitivity. |

Communication: Promotes effective communication with patients, families, and other health professionals

| Critical Deficiency – Level 1 | Variable Skills –  Level 2 | Effective Skills –  Level 3 | Exemplary Skills – Level 4 |
| --- | --- | --- | --- |
| Use standard medical interview template to engage all patients regardless of unique socioeconomic, cultural, and physical needs. Does not effectively engage other health professionals. | Attempts to identify unique aspects of each patient and use them to establish an effective physician-patient alliance. Approaches all healthcare professionals in the same way, regardless of their role in patient care. | Systematically identifies the unique needs of each patient and utilizes them to build a strong physician-patient relationship. Effectively communicates with other healthcare providers with an understanding of their role in patient care. | Effortlessly identifies the unique needs of each patient and builds an authentic relationship with them and their support system. Seamlessly broaches sensitive topics in a way that puts patients at ease. Approaches other healthcare providers as individuals to build a working relationship that provides the best outcomes for the patient. |

Teaching Style: Establishes positive learning climate

| Critical Deficiency – Level 1 | Variable Skills –  Level 2 | Effective Skills –  Level 3 | Exemplary Skills – Level 4 |
| --- | --- | --- | --- |
| Performs little education, does not encourage resident participation in academic discussions. | Performs didactic teaching but teaching sessions are not tailored to residents level of training. May ask for resident opinions with limited discussion. | Solicits resident opinions and discusses their merits on a basic level. Willing to teach complex topics. Tailors teaching to residents level of training. Provides guidance of future topics to study. | Encourages residents to share opinions and provide individualized teaching based on resident competency level. Provides the tools and motivation necessary for residents formulate essential questions and to self-teach complex topics. |
